# Association of MTDH immunohistochemical expression with metastasis and prognosis in female reproduction malignancies: a systematic review and meta-analysis

**DOI:** 10.1038/srep38365

**Published:** 2016-12-05

**Authors:** Yongbin Hou, Lihua Yu, Yonghua Mi, Jiwang Zhang, Ke Wang, Liyi Hu

**Affiliations:** 1 Department of clinical laboratory, Affiliated Yongchuan Hospital of Chongqing Medical University, Chongqing 402160, China; 2 Department of CIK treatment laboratory, Affiliated Yongchuan Hospital of Chongqing Medical University, Chongqing 402160, China

## Abstract

Various literatures have demonstrated that overexpression of Metadherin (MTDH) is correlated with tumor metastasis and it can predict poor survival outcomes in female reproduction malignancies. In order to enhance the statistical power and reach a recognized conclusion, we conducted a systematic review and meta-analysis to thoroughly investigate the association of MTDH expression with tumor metastasis and survival outcomes following PRISMA guidelines. Odds ratios (ORs) and hazard ratios (HRs) were used to demonstrate the impact of MTDH on tumor metastasis and prognosis respectively. Data were pooled with appropriate effects model on STATA12.0. Our results indicated that high MTDH expression is significantly correlated with higher mortality for breast, ovarian and cervical cancer. High immunohistochemical expression of MTDH is remarkably associated with shorter disease-free survival (DFS) in breast cancer but not in ovarian cancer. The pooled results suggested that high level of MTDH significantly predicted distant metastasis and lymph node metastasis in breast cancer. Strong associations were observed between MTDH expression and lymph node metastasis in ovarian and cervical cancer. In conclusion, MTDH might be a novel biomarker which can effectively reflect metastasis status and prognosis of breast cancer. However, its application in clinical practice needs more prospective studies with large samples.

Reproduction malignancies, including breast cancer, cervical cancer, ovarian cancer and endometrial cancer, have been one of the major causes of death in females, among which, breast cancer is the most common malignancy and the leading cause of cancer death in western countries. With an increasing incidence, reproduction malignancies have seriously affected living quality and health of the patients. It is estimated that there were 1.7 million new cases, causing 521,900 deaths throughout the world in 2012^1^. In China, with 268.6 thousand new cases, breast cancer accounts for 15% of all the new cases of cancers in 2015. Moreover, cervical and ovarian cancers are predicted to cause 30.5 thousand and 22.5 thousand deaths in Chinese female respectively according to the latest cancer statistics^2^. Although nowadays medical treatment is advanced, the prognosis of reproduction malignancies patients is dismal, and metastasis is still the major cause of death. In addition, there lacks appropriate indicator which can effectively predict the prognosis of reproduction malignancies patients. Therefore, it is urgent to seek an impeccable marker which can reflect the status of metastasis and clinical survival outcomes for patients with reproduction malignancies.

Recently, researchers have made great efforts to explore new biomarkers which are associated with the diagnosis, progression and prognosis of reproduction malignancies. Metadherin (MTDH), as a cell surface protein, could induce breast cancer cells transferring to lung in mouse model. Its gene, astrocyte elevated gene-1 (AEG-1), was first cloned in human fetal astrocytes as an inducible gene by human immunodeficiency virus 1 (HIV)-1 and tumor necrosis factor-α (TNF-α) in 2002^3^,^4^.Subsequently, clinical trial demonstrated that MTDH is a novel prognostic biomarker and high MTDH expression is associated with tumor progression and short overall survival (OS) time in breast cancer^5^. Similar results were found in other reproduction malignancies, such as epithelial ovarian cancer^6^, cervical cancer^7^ and endometrial cancer^8^. Two authors have performed meta-analysis to evaluate the clinicopathological and prognostic role of MTDH in squamous cell carcinoma and gastrointestinal cancers respectively. They concluded that high MTDH expression was remarkably correlated with lymph node metastasis, distant metastasis and short OS^9^,^10^. A review has extensively described the pleiotropic roles of MTDH in breast cancer^11^. Collectively, accumulating evidence suggested that MTDH might participate in the tumor metastasis process and can be regarded as therapeutic target of reproduction malignancies.

Up till now, no quantitative evaluation was performed. Because of the limited sample size, the conclusion of a single study lacks power of reliability. Hence, by reviewing published literature we performed a comprehensive meta-analysis in order to get a consistent and reliable conclusion and to cast light on the impact of MTDH expression on metastasis and survival status.

## Results

### Literature identification and selection

**Figure 1:**
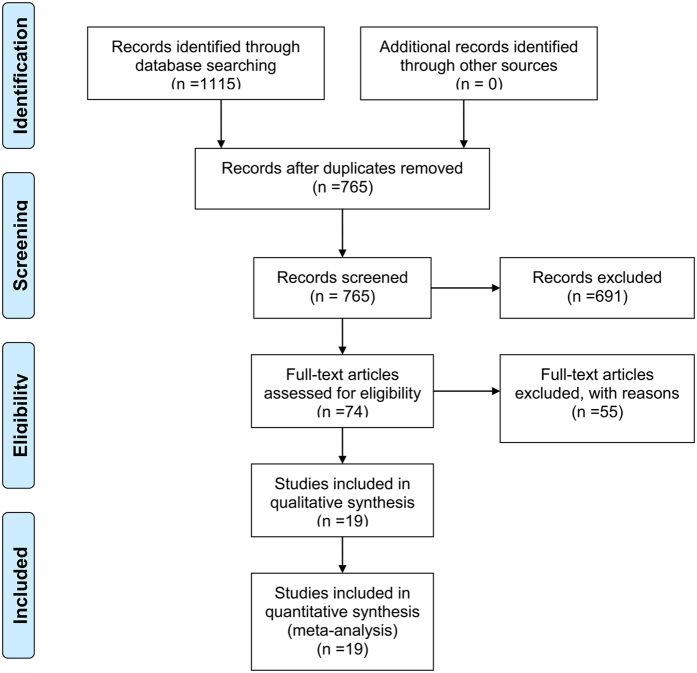
Flow diagram of the current systematic review and meta-analysis.

In total, 1115 studies (791 in English and 324 in Chinese) were retrieved for our systematic review and subsequent meta-analysis after searching different databases. After removing duplicates, titles and abstracts of the remained 765 papers were prudently screened, among which 74 studies were obtained as they evaluated the role of MTDH/LYRIC/AEG-1/3D3 in female reproduction malignancies. Meanwhile, a total of 55 potentially relevant papers were excluded as they were against the inclusion criteria of our meta-analysis, of which three studies only investigated the gene expression of MTDH in breast and ovarian cancer. Two studies^12^,^13^ which only provided survival curve but failed to calculate HR value were excluded from meta-analysis. They were included in the systematic review. Another 50 studies were excluded for lacking survival data or using cell lines to explore the impact of MTDH on biological characteristics, mechanisms and pathways of reproduction malignancies. Finally, a total of 19 studies (17 English articles and 2 Chinese articles)^5^,^6^,^7^,^8^,^14^,^15^,^16^,^17^,^18^,^19^,^20^,^21^,^22^,^23^,^24^,^25^,^26^,^27^,^28^ published from 2008 to 2016 were included in our meta-analysis (Fig. 1).

### Characteristics of included literature

The 19 studies included in our meta-analysis analyzed the correlation of MDTH levels with clinicopathological parameters and survival outcomes in 2483 female reproduction malignancies patients. Among them 8 studies were about breast cancer^5^,^14^,^15^,^16^,^21^,^23^,^25^,^28^, while 7 were about ovarian cancer^6^,^17^,^18^,^20^,^22^,^26^,^27^, 3 about cervical cancer^7^,^19^,^24^ and 1 about endometrial cancer^8^.

A large proportion of these trails were performed in China and the number of patients ranged from 60 to 225. As for inclusion criteria, all the samples of included studies were tissues and their MTDH expression was examined by immunohistochemical (IHC). Eleven studies^5^,^6^,^8^,^15^,^16^,^17^,^18^,^21^,^23^,^25^,^26^ defined staining index (SI) ≥ 4 as cut-off value of positive expression while the other 8 studies^7^,^14^,^19^,^20^,^22^,^24^,^27^,^28^ did not. The follow-up time ranged from 36 to 180 months.

Data of distant metastasis were available in 8 studies^5^,^14^,^15^,^16^,^17^,^19^,^23^,^25^,^28^ and data of lymph node metastasis were found in 12 studies^5^,^7^,^8^,^15^,^16^,^17^,^19^,^23^,^25^,^26^,^27^,^28^. For survival analysis, 14 studies^5^,^6^,^7^,^8^,^14^,^16^,^17^,^18^,^20^,^23^,^24^,^25^,^26^,^27^ examined the association of MTDH expression with mortality and 8 studies^6^,^8^,^16^,^20^,^24^,^25^,^27^,^28^ were available for disease-free survival (DFS). The concomitant variables of multivariate analysis were list in Table 2. Further details on characteristics of the included studies were shown in Table 1 and Table 2.

### Meta-analysis

#### Breast cancer

**Figure 2:**
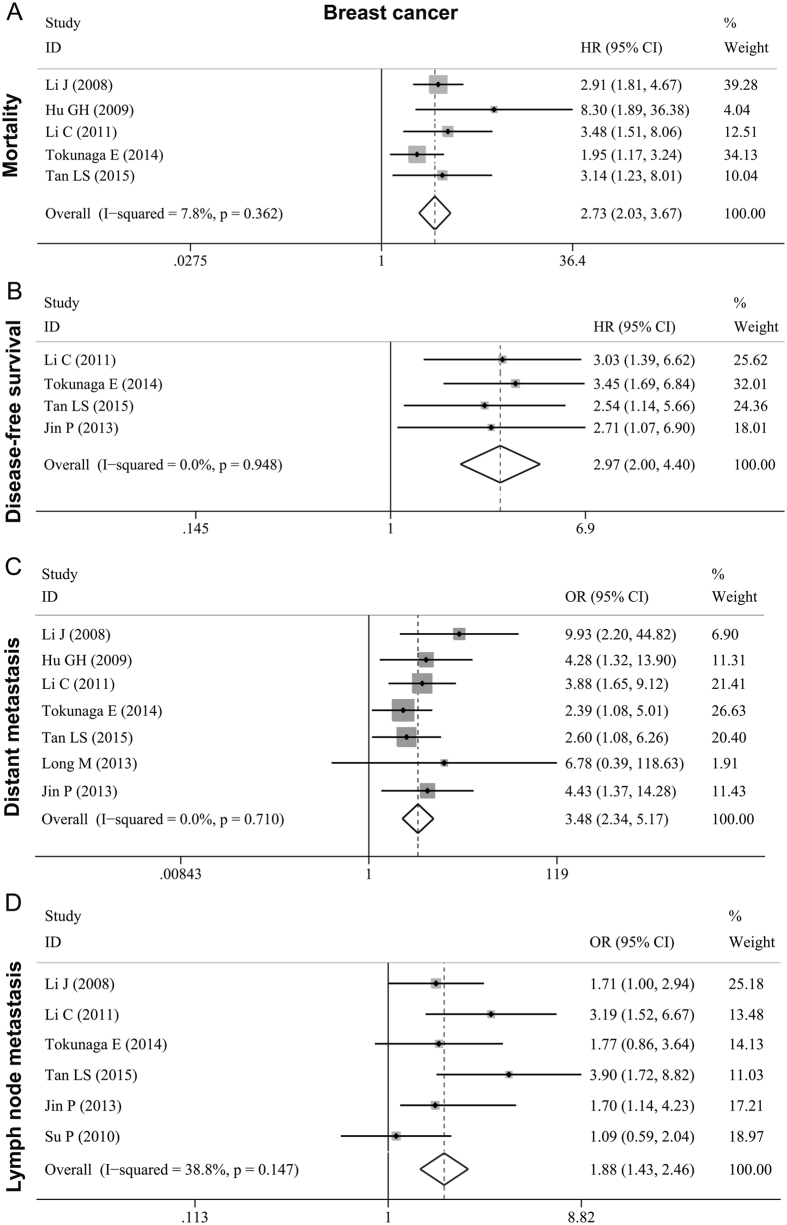
Forest plots of meta-analysis for the association between MTDH and metastasis as well as prognosis in breast cancer. (**A**) mortality; (**B**) DFS; (**C**) distant metastasis; (**D**) lymph node metastasis.

Eight studies^5^,^14^,^15^,^16^,^21^,^23^,^25^,^28^ with a total of 1167 breast carcinoma cases examined the relationship between MTDH and metastasis as well as survival status. Five studies^5^,^14^,^16^,^23^,^25^ presented mortality information on MTDH expression, with a pooled HR of 2.728 (95% CI: 2.027–3.671, *P* < 0.001) (Fig. 2A, Table 3). As their heterogeneity was weak (*I*^*2*^ = 7.8%, *P* = 0.362), the fixed effects model was applied. Four studies^16^,^23^,^25^,^28^ assessed the association of MTDH with DFS. Its pooled HR was 2.966 (95% CI: 1.997–4.405, *P* < 0.001) (Fig. 2B, Table 3) and no evidence of heterogeneity was found (*I*^*2*^ = 0.0%; *P* = 0.948). Seven studies^5^,^14^,^16^,^19^,^23^,^25^,^28^ reported data on MTDH expression and distant metastasis in breast cancer. The pooled OR was 3.480(95% CI: 2.342–5.170, *P* < 0.001) (Fig. 2C, Table 3), and the heterogeneity was not significant (*I*^*2*^ = 0.0%; *P* = 0.710). Six studies^5^,^15^,^16^,^23^,^25^,^28^ examined the correlation of MTDH with lymph node metastasis. Moderate heterogeneity was observed (*I*^*2*^ = 38.8%; *P* = 0.147) and the pooled fixed effects HR was 1.880 (95% CI: 1.433–2.465; *P* < 0.001) (Fig. 2D, Table 3). The above results indicated that high MTDH expression can lead to tumor metastasis and poor prognosis for females who suffered breast cancer.

#### Ovarian cancer

**Figure 3:**
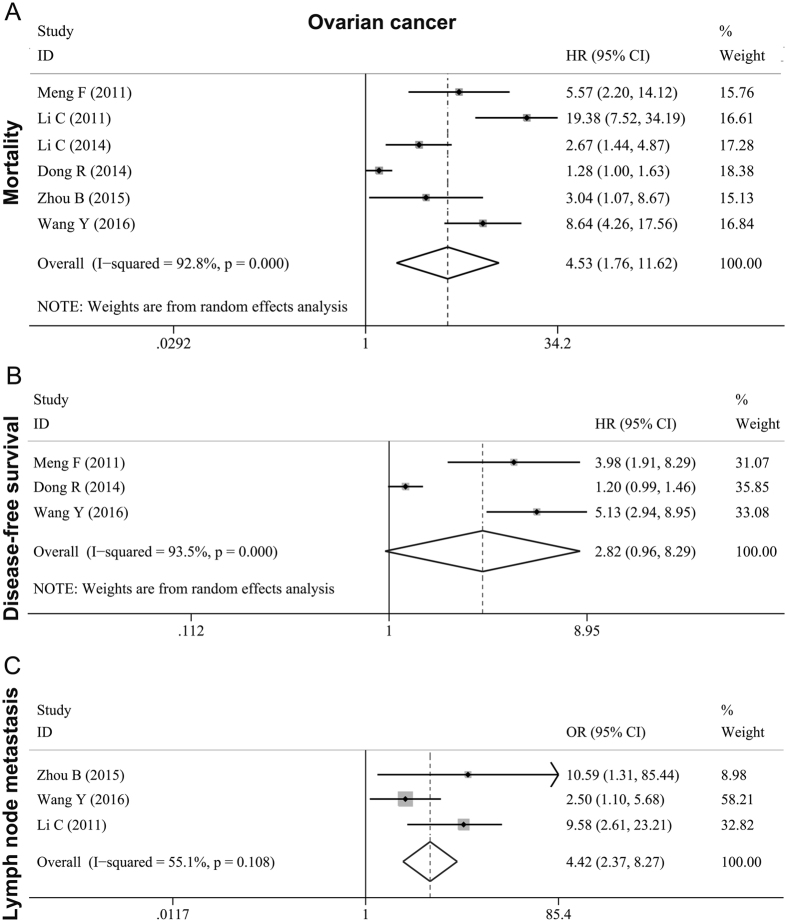
Forest plots of meta-analysis for the association between MTDH and metastasis as well as prognosis in ovarian cancer. (**A**) mortality; (**B**) DFS; (**C**) lymph node metastasis.

Seven studies^6^,^17^,^18^,^20^,^22^,^26^,^27^, with a total of 514 female cases, concluded that MTDH was a poor predictor for ovarian cancer. Six studies^6^,^17^,^20^,^22^,^26^,^27^ with mortality data had a high heterogeneity of (*I*^*2*^ = 92.5%; *P* < 0.001), and the pooled HR was 4.525(95% CI: 1.763–11.618, *P* < 0.001) (Fig. 3A, Table 3). Therefore random effects model was used. The pooled HR was 2.817(95% CI: 0.957–8.292, *P* = 0.060) (Fig. 3B, Table 3) for those 3 studies^6^,^20^,^27^ which described the DFS data. Significant high heterogeneity was observed and a random effects model was also used (*I*^*2*^ = 93.5%; *P* < 0.001) for these 3 studies. There were another 3 studies^17^,^26^,^27^ with 336 ovarian cancer cases examining the association of MTDH overexpression with lymph node metastasis. The pooled effects OR was 5.328 (95% CI: 1.870–15.175, *P* = 0.002) (Fig. 3C, Table 3). Since the inter-study heterogeneity was moderate (*I*^*2*^ = 55.1%; *P* = 0.108), we applied the random-effects model.

#### Cervical cancer

**Figure 4:**
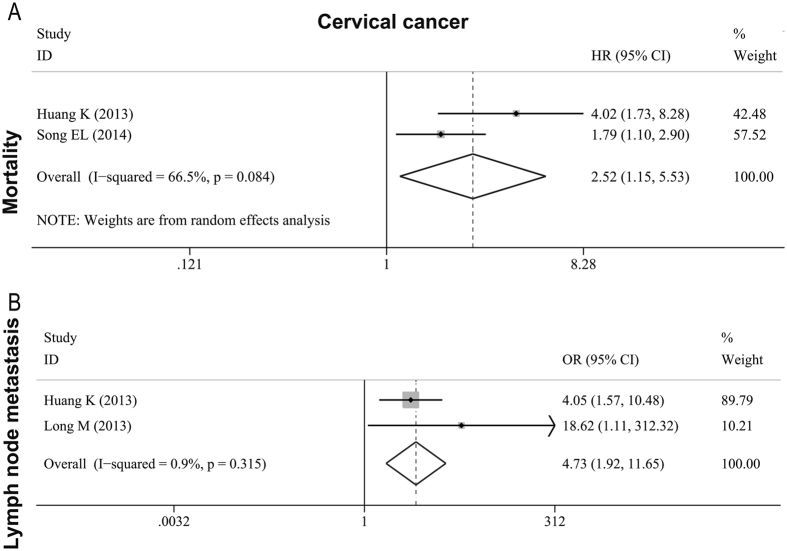
Forest plots of meta-analysis for the association between MTDH and metastasis as well as prognosis in cervical cancer. (**A**) mortality; (**B**) lymph node metastasis.

Three studies^7^,^19^,^24^ with a number of 390 female cases described MTDH as the predictor of tumor deterioration in cervical cancer. Two of the studies^7^,^24^ stated a high mortality for patients with an overexpression of MTDH and we calculated a combined HR. The result was 2.524 (95% CI: 1.152–5.529, *P* < 0.001) (Fig. 4A, Table 3). However, the inter-study heterogeneity was moderate (*I*^*2*^ = 66.5%; *P* = 0.084), thus we performed the random-effects model. To examine the relationship between high tumoral MTDH expression and lymph node metastasis in cervical cancer, we combined OR from two studies^7^,^19^ and the pooled OR was 4.732 (95% CI: 1.922–11.652, *P* = 0.001) (Fig. 4B, Table 3). No significant inter-study heterogeneity was observed (*I*^*2*^ = 0.9%; *P* = 0.315).

#### Meta-analysis of all the included cancers

Based on 14 studies that examined the association of MTDH with mortality of various female reproduction cancers, the pooled random effects HR was 3.647 (95% CI: 2.385–5.577, *P* < 0.001) (Fig. S1A, Table 3). There was significant high heterogeneity when all studies were combined (*I*^*2*^ = 83.9%; *P* < 0.001). A total of 8 studies^6^,^8^,^16^,^20^,^23^,^25^,^27^,^28^ evaluated the relevance of MTDH expression with DFS in female reproduction cancers, with the overall random effects HR of 2.917(95% CI: 1.715–4.963, *P* < 0.001) (Fig. S1B, Table 3). The heterogeneity was high (*I*^*2*^ = 83.9%; *P* < 0.001). The pooled results from these studies indicated MTDH overexpression in reproduction cancers can cause a short lifespan for patients. Besides, 8 studies^5^,^14^,^16^,^17^,^19^,^23^,^25^,^28^ examined the association of MTDH with distant metastasis and 12 studies^5^,^7^,^8^,^15^,^16^,^17^,^19^,^23^,^25^,^26^,^27^,^28^ examined the association of MTDH with lymph node metastasis. The overall fixed effects OR was 3.739 (95% CI: 2.558–5.466, *P* < 0.001) (Fig. S1C, Tables 3) and 2.696 (95% CI: 1.874–3.879, *P* < 0.001) (Fig. S1D, Table 3), respectively. Both pooled results showed that MTDH in female reproduction cancers can be regarded as an unfavorable predictor in tumor metastasis. No significant heterogeneity was found when examining the relationship between MTDH and distant metastasis (*I*^*2*^ = 0.0%; *P* = 0.619), while moderate heterogeneity can be found in the studies related to lymph node metastasis (*I*^*2*^ = 53.2%; *P* = 0.015).

### Sensitivity analysis

After excluding every single study in order, the pooled HRs for mortality and ORs metastasis did not change significantly (Fig. S2A,C,D). For DFS, the study published by Dong R^20^ *et al*. significantly affected the pooled HR (Fig. S1B). After eliminating the study published by Dong R^20^ *et al*., the pooled HR changed from 2.641 (95% CI: 1.497–4.658, *P* = 0.001) to 3.300 (95% CI: 2.338–4.657, *P* < 0.001) (Fig. S3) and no heterogeneity was observed. For multiple cut-off value used in different studies, we conducted a sensitivity analysis including only 8 studies which used the same cut-off value (SI ≥ 4) for mortality analysis. The result was significant with an HR of 3.613 (95% CI: 2.806–4.653, *P* < 0.001).

### Publication bias

**Figure 5:**
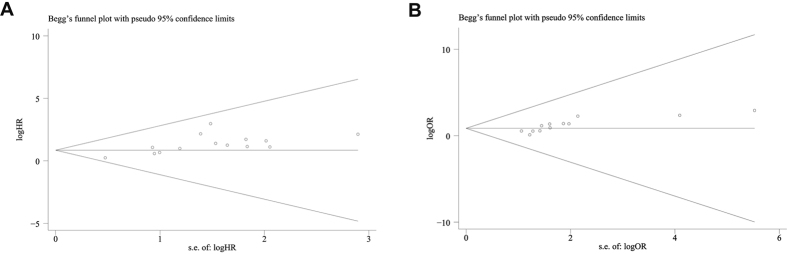
Funnel plots for mortality and lymph node metastasis group of included studies. (**A**) mortality; (**B**) lymph node metastasis.

For the group with more than 10 studies, we performed Begg’s test to assess publication bias. Our results showed that there was no evidence of publication bias in mortality (Fig. 5A, *P* = 0.112). However, significant publication bias was observed for the studies concerned with lymph node metastasis (Fig. 5B, *P* = 0.001). Trim and fill method was conducted to enroll missing studies. After enrolling missing studies, the pooled random-effects OR was 2.150 (95% CI: 1.465–3.155) (Fig. S4). Due to the limited number of studies, publication bias was not examined for other group of meta-analysis.

### Systematic review

Two studies^12^,^13^ provided survival curve but failed to calculate HR value were included in systematic review. Ward A^13^ *et al*. showed that high MTDH expression was remarkably associated with short DFS in breast cancer (*P* = 0.0233) with a cohort of patients from public data (GSE1378). The study published by Liu P^12^ *et al*. suggested that high MTDH level was significantly correlated with poor OS in triple negative breast cancer (*P* = 0.006), but no statistical significance was observed for DFS (*P* = 0.065).

## Discussion

Meta-analysis can enhance statistical power by quantitatively combining the results of multiple independent studies. As several studies have regarded MTDH as the potential biomarker which can indicate the metastasis and prognosis in malignancies, and as MTDH could miraculously induce breast cancer cells transferring to lung in mouse model, we carried out this comprehensive systematic review and meta-analysis based on published literature to thoroughly investigate the significance of MTDH in reproduction malignancies. Our results showed that MTDH, as a cell surface protein, is significantly associated with the mortality of patients with reproduction malignancies (HR = 3.647), including breast cancer (HR = 2.728), ovarian cancer (HR = 4.525), cervical cancer (HR = 2.524) and endometrial cancer (HR = 2.524). For DFS, significant result was observed in breast cancer (HR = 2.966) but not in ovarian cancer. Moreover, high MTDH expression remarkably increased the risk of distant metastasis (HR = 3.739) and lymph node metastasis (HR = 2.696) in reproduction malignancies. A strong association was observed between MTDH and metastasis as well as prognosis in clinical malignancies. The pooled results of those 8 studies^5^,^14^,^15^,^16^,^21^,^23^,^25^,^28^ in breast cancer enrolled in our meta-analysis showed that high MTDH is remarkably correlated with high risk of metastasis and poor prognosis. Meanwhile, no significant heterogeneity was observed (P > 0.05). According to the above evidences, MTDH is likely to be the fundamental material for tumor metastasis and can lead to poor prognosis in reproduction malignancies. For breast cancer, the results of our meta-analysis implied that MTDH might be a novel biomarker being applied to clinic.

Heterogeneity is one of possible factors impacting the pooled results of meta-analysis^29^. Heterogeneity was observed when we mixed all reproduction malignancies and calculated the combined effects of MTDH on mortality (*I*^*2*^ = 82.1%) and DFS (*I*^*2*^ = 80.7%). However, when we divided the included studies into different cancer types, no significant heterogeneity was found in breast cancer (P > 0.05). Thus, the difference in cancer type might be one of the potential factors that caused inter-study heterogeneity. For ovarian cancer, the possible reasons of heterogeneity might be the selection of tissue and the computing method of HR. Of 7 studies in ovarian cancer, 3 studies^6^,^17^,^27^ only selected epithelial ovarian cancer while other 4 studies^18^,^20^,^22^,^26^ contained various types of ovarian cancer. Li C^18^ *et al*. only analyzed the prognosis of patients with stages III–IV. The study published by Dong R^20^ *et al*. only provided survival curve while the other studies offered HR value of multivariate analysis. To take all the heterogeneity described above into consideration, we pooled the HRs using random effects model. For the combination of those studies which didn’t have statistically significant heterogeneity, a fixed effects model was used to acquire more accurate combined HR.

The unpublished studies or studies with negative results were not included in our meta-analysis, which might cause same bias and affect the pooled results. For publication bias, we performed Begg’s test in the mortality and lymph node metastasis group. No obvious publication bias (*P* = 0.112) was observed in mortality group while significant publication bias was observed in lymph node metastasis group (*P* = 0.001). Thus, trim and fill method was conducted to enroll missing studies. However, the final result was not distinctly altered, which implied that the pooled result was stable and creditable. The results of sensitivity analysis indicated that our meta-analysis was stable for all the groups except DFS. For DFS, the result didn’t change obviously after eliminating the study published by Dong R^20^ *et al*. Meanwhile, the result of sensitivity analysis for studies using the same cut-off (SI ≥ 4) was similar to all studies of mortality group, which implied the reliability of our conclusion.

The current meta-analysis featured the following strengths. Firstly, we conducted a comprehensive search and extracted up-to-date published literatures. Secondly, all the included studies were with sufficient patients ( > 50), which favorably avoided the effects of small sample. Thirdly, all the samples of the included studies were from tissues and their MTDH was examined by IHC, which ensured the homogeneity of our meta-analysis. Moreover, there was no significant heterogeneity when we pooled the studies in breast cancer. The strengths above immensely enhanced the reliability of our meta-analysis. Still, there were also a few limitations. Four studies^23^,^24^,^25^,^28^ only provided survival cure rather than HR value, thus we had to extract HR value using the software Engauge Digitizer version 4.1, which might cause bias to accurate HR value. Meanwhile, different variables adjusted by multivariate analysis of each single study also affected the pooled HRs.

Recently, a number of studies have investigated the impact of MTDH in reproduction malignancies. They rifely concluded that MTDH was associated with specific biological characteristics and molecular pathways related to tumor deterioration, but the exact mechanism was still unclear. Chemoresistance/radioresistance might be one of the reasons related to poor prognosis. MTDH could mediate drug resistance in various cancers^30^ as suggested by Meng *et al*. For reproduction malignancies, it was reported that inhibition of MTDH increases cancer cells’ sensitivity to chemotherapy drugs, such as AZD6244, tumor necrosis factor-a-related apoptosis-inducing ligand (TRAIL) and HDAC inhibitor LBH589^31^,^32^,^33^. Interestingly, Zhao Y *et al*. confirmed that MTDH induces radioresistance and inhibits apoptosis in cervical cancer cells^34^. MTDH also acts as a regulator for tumor progression and high MTDH expression promotes the proliferation, invasion and metastasis of cancer cell in reproduction malignancies by interacting with staphylococcal nuclease domain-containing 1, Ha-ras protein or RNA^34^,^35^,^36^,^37^,^38^,^39^. According to the published studies, MTDH might exert its powerful regulatory functions in reproduction malignancies via molecular pathways: PI3K-Akt^37^, protective autophagy^40^, NF-κB^36^,^41^,^42^ and PTEN/AKT^21^,^43^.Collectively, MTDH plays a vital role in tumor progression. Thus, down-regulating of MTDH might be an effective method to lengthen survival time and reduce mortality in reproduction malignancies.

In conclusion, our meta-analysis suggests that high MTDH expression increases risk of distant metastasis and lymph node metastasis in reproduction malignancies. Furthermore, high MTDH expression is remarkably associated with high mortality in reproduction malignancies. Specifically, the results our meta-analysis indicates that high MTDH expression is strongly associated with high risk of tumor metastasis and worse prognosis in breast cancer. Thus, our study provides evidence that MTDH might be a potential novel biomarker effectively reflecting metastasis status and prognosis of breast cancer patients, which might help to formulate a better therapy for individual patient. Simultaneously, to enhance the creditability of our meta-analysis, more prospective studies with large samples are needed.

## Methods

### Literature search strategy and selection

A comprehensive electronic literature search was conducted using the following English databases and Chinese databases: PubMed, Wiley Online Library, Science Direct, EMBASE, Cochrane Central Register of Controlled Trials, ISI Web of Science, China National Knowledge Infrastructure (CNKI) and Wanfang database. The literature search strategy was as follow: “metadherin OR MTDH OR astrocyte elevated gene-1 OR AEG1 OR AEG-1 OR LYRICOR LYRIC/3D3 OR 3D3″[All Fields] AND “tumor OR tumour OR cancer OR carcinoma OR neoplasm OR neoplastic OR malignancy”[All Fields] AND “prognosis OR prognostic OR survival OR survivance OR mortality OR outcome OR predict* OR follow-up” [All Fields]. The search was not finished until September 21, 2016.

Firstly, all the titles and abstracts of each literature found by the search strategy were imported to EndNote X7. Then, two independent authors (Yongbin Hou and Liyi Hu) screened out the eligible literature which investigated the association between MTDH/LYRIC/AEG-1/3D3 expression and clinicopathological features as well as survival outcomes in patients with female reproduction cancers. Finally, full-text articles of eligible literatures were reviewed in detail, including references cited in the literature, according to our inclusion and exclusion criteria.

The detailed inclusion criteria were listed in Table 4. The exclusion criteria were listed as follow: (1) animal or cell line trails, case reports, reviews and meta-analysis; (2) studies failed to estimated HRs/ORs and its 95% CI. In addition, we attempted to contact authors, if possible, to obtain raw data for the studies whenever inadequate data were provided to estimate HRs/ORs and its 95% CI. For the studies including overlapped patients, we only included the most complete studies in our meta-analysis. If there were controversies, the two authors would solve the problem by discussion.

### Data extraction

The following information was extracted from each study by two independent authors (Yonghua Mi and Yongbin Hou) using a standard excel form: first author; year of publication; types of cancer; source and number of patients; detected method for MTDH/LYRIC/AEG-1/3D3; cut-off value for positive MTDH/LYRIC/AEG-1/3D3 expression; longest follow-up time. For metastasis status of included studies, ORs as well as their 95% CI were extracted. For survival analysis, HRs along with their 95% CI and P value were extracted. If the results multivariate analysis were available, the HRs/ORs values of multivariate analysis and concomitant variables were extracted. Otherwise, the HRs/ORs of univariate analysis (univariate cox regression/logistic regression, survival curve or available data to estimate HR/OR) were extracted. Subsequently, all the data were double-checked by a third author (Liyi Hu) and controversies were solved by discussion.

### Statistical analysis

For the meta-analysis, we stratified the results by the type of reproduction carcinoma. To describe the intensity of relationship between MTDH and clinical outcomes in reproduction malignancies patients, HR value and OR value as well as their 95% CI were used. With a standard excel form, the HR value and OR value of each single study were extracted. For HR and OR value, we extracted the relevant data directly if they were given in the studies. As for those statistical variables which were indirectly stated, we estimated their values on the basis of available data or Kaplan-Meier curves by methods of Jayne F Tierney^44^ described.

Subsequently, the HRs (ORs) values were quantificationally combined on STATA12.0 (STATA Corp., College,Texas). For heterogeneity analysis, the Cochran Q test and the Higgins *I*^*2*^ statistical method^29^ were carried out. When statistically significant heterogeneity was observed (*P* < 0.05), the random-effects model^45^ was used to combine relevant data. Otherwise, the fixed-effects model^46^ was used (when *P* > 0.05). The *I*^*2*^ statistic was regarded as a quantitative measure of the degree of inconsistency among the studies. When *I*^*2*^ = 0, there was no heterogeneity. The larger the percentage, the increasing heterogeneity^47^. As for the consistency of our presentation, an *I*^*2*^ value of 1 to 25%, 25 to 75%, 75to 100% was considered low heterogeneity, moderate heterogeneity and high heterogeneity, respectively. A statistically significant HR (OR) > 1 suggested that high MTDH indicated worse survival outcomes and increased the risk of metastasis. *P* values less than 0.05 were considered to be statistically significant.

Concerning the positive results being more likely to published, publication bias was assessed by Begg’s test with funnel plots when the meta-analysis included 10 or more studies. A symmetrical inverted funnel implied that there was no significant publication bias among included studies. Trim and fill method was conducted to reduce publication bias if statistically significant publication bias was observed. Furthermore, sensitivity analysis was conducted by eliminating single study successively and the rest studies was pooled to examine the stability of the results. Also, sensitivity analysis was performed in studies of mortality which employed the same cut-off value (SI ≥ 4). The current study is a meta-analysis which gained ethical approval and all the procedures of this study followed PRISMA guidelines^48^.

## Additional Information

**How to cite this article**: Hou, Y. *et al*. Association of MTDH immunohistochemical expression with metastasis and prognosis in female reproduction malignancies: a systematic review and meta-analysis. *Sci. Rep.* **6**, 38365; doi: 10.1038/srep38365 (2016).

**Publisher's note:** Springer Nature remains neutral with regard to jurisdictional claims in published maps and institutional affiliations.

## Supplementary Material

Supplementary Information

## Figures and Tables

**Table 1:** Main characteristic of included studies.

Author	Year	Cancer types	Source of patients	Cases	Samples	Method	Cut-off value for positive	Follow-up (Months)
Li J^5^	2008	breast cancer	China	225	tissue	IHC	SI ≥ 4	80*
Hu GH^14^	2009	breast cancer	America	170	tissue	IHC	NR	180*
Su P^15^	2010	breast cancer	China	162	tissue	IHC	SI ≥ 4	/
Li C^16^	2011	breast cancer (triple-negative)	China	125	tissue	IHC	SI ≥ 4	70*
Tokunaga E^23^	2014	breast cancer	Japan	195	tissue	IHC	SI ≥ 4	119
Du C^21^	2014	breast cancer	China	118	tissue	IHC	SI ≥ 4	/
Tan LS^25^	2015	breast cancer (triple-negative)	China	112	tissue	IHC	SI ≥ 4	90
Meng F^6^	2011	epithelial ovarian cancer	China	81	tissue	IHC	SI ≥ 4	78
Li C^17^	2011	epithelial ovarian cancer	China	157	tissue	IHC	SI ≥ 4	/
Li C^18^	2012	ovarian cancer	China	101	tissue	IHC	SI ≥ 4	96
Li C^22^	2014	ovarian cancer	China	102	tissue	IHC	SI ≥ 3	>36
Dong R^20^	2014	ovarian cancer	China	76	tissue	IHC	SI ≥ 2	60
Zhou B^26^	2015	ovarian cancer	China	73	tissue	IHC	SI ≥ 4	80
Long M^19^	2013	cervical cancer	China	220	tissue	IHC	SI ≥ 3	/
Huang K^7^	2013	cervical cancer	China	90	tissue	IHC	positive rate ≥ 40%	80*
Song EL^24^	2014	cervical cancer	China	80	tissue	IHC	intensity ≥ 17.4%	60*
Song HT^8^	2010	endometrial cancer	China	174	tissue	IHC	SI ≥ 4	76
Wang Y^27^	2016	ovarian cancer	China	162	tissue	IHC	SI ≥ 3	78
Jin P^28^	2013	breast cancer	China	60	tissue	IHC	SI ≥ 2	125*

^*^Extracted from survival curve; IHC: immunohistochemistry; SI: staining index = staining intensity × proportion of positive tumor.

**Table 2:** The HRs/ORs value and concomitant variables of included studies.

Author	Year	Distant metastasis (OR/HR)	lymph node metastasis (OR/HR)	Survival analysis	HR (95% CI)	P	Concomitant variables
Li J^5^	2008	9.930 (2.200–44.820)	1.714 (0.999–2.943)	OS (M)	2.906 (1.810–4.668)	<0.001	T classification, N classification.
Hu GH^14^	2009	4.280 (1.320–13.900) (M)	/	CSS (U)	8.300 (1.894–36.376)	0.005	ER, PR, HER2, p53 and tumor size.
Su P^15^	2010	/	1.094 (0.587–2.038)	/	/	/	
Li C^16^	2011	3.875 (1.647–9.118)	3.188 (1.524–6.671)	OS (M)	3.484 (1.505–8.062)	<0.001	tumor size, lymphatic and venous invasion, lymph node status.
				DFS (M)	3.032 (1.388–6.622)	0.014	
Tokunaga E^23^	2014	2.39 (1.08–5.01) (M)	1.771 (0.861–3.642)	DFS (M)	3.450 (1.690–6.840)	0.001	tumor size, nodal involvement, nuclear grade, lymphovascular invasion, ER, PR, HER2.
				OS (U)^a^	1.950 (1.173–3.241)	0.01	
Tan LS^25^	2015	2.604 (1.084–6.257)	3.900 (1.724–8.821)	OS (U)^a^	3.140 (1.230–8.010)	0.017	
				DFS (U)^a^	2.540 (1.140–5.660)	0.023	
Meng F^6^	2011	/	/	OS (M)	5.573 (2.199–14.124)	<0.001	FIGO stage, residual tumor, histological type.
				DFS (M)	3.982 (1.913–8.286)	<0.001	
Li C^17^	2011	8.541 (2.561–37.461) (M)	9.581 (2.613–23.214) (M)	/	/	/	FIGO stage, residual tumor size.
Li C^18^	2011	/	/	OS (M)	19.380 (7.518–34.192)	<0.001	age, lymph node metastasis, histopathological differentiation, serum CA-125 level, cytoreduction, FIGO stage, residual tumour size, chemotherapy resistance.
Li C^22^	2014	/	/	OS (M)	2.673 (1.445–4.867)	0.002	age, primary malignancies, extent of disease, ascites, size, time to diagnosis.
Dong R^20^	2014	/	/	OS (U)^a^	1.280 (1.004–1.631)	0.046	
				DFS (U)^a^	1.200 (0.989–1.456)	0.065	
Zhou B^26^	2015	/	10.588 (1.312–85.438)	OS (M)	3.037 (1.067–8.670)	0.036	age, histological type, differentiation degree, lymph node metastasis, clinical stage.
Huang K^7^	2013	/	4.050 (1.565–10.481)	OS (M)	4.021 (1.734–8.283)	0.027	age, tumor size, histological classification, clinical stage, pathological grade, lymph node metastasis.
Song EL^24^	2014	/	/	OS (U)^a^	1.790 (1.105–2.900)	0.018	
Long M^19^	2013	6.781 (0.388–118.631)	18.616 (1.110–312.323)	/	/	/	
Song HT^8^	2010	/	3.929 (1.437–10.743)	OS (M)	4.960 (1.774–13.869)	0.002	FIGO stage, lymphatic and venous invasion, histological type.
				DFS (M)	3.573 (1.499–8.518)	0.004	
Wang Y^27^	2016	/	2.500 (1.101–5.676)	OS (M)	8.644 (4.255–17.556)	<0.001	FIGO stage, residual tumor.
				DFS (M)	5.132 (2.943–8.949)	<0.001	
Jin P^28^	2013	4.429 (1.373–14.283)	1.699 (1.145–4.230)	DFS (U)^a^	2.710 (1.070–6.900)	0.036	

HR: hazard ratio; OR: odds ratios; OS: overall survival; CSS: cancer-specific survival; DFS: disease-free survival; M: multivariate analysis; U: univariate analysis;

^a^extracted from survival curve; ER: estrogen receptor; PR: progesterone receptor; HER2: human epidermal growth factor receptor-2; FIGO: International Federation of Gynecology and Obstetrics.

**Table 3:** Meta-analysis of HRs/ORs evaluating the association of MTDH with tumor metastasis and prognosis.

Groups	Studies	Pooled HR/OR (95% CI)	*P*	Effect model	Heterogeneity
***Mortality (OS + CSS)***					
Overall	14	3.647 (2.385–5.577)	<0.001	random	*I*^*2*^ = 83.9%; *P* < 0.001
Breast cancer	5	2.728 (2.027–3.671)	<0.001	fixed	*I*^*2*^ = 7.8%; *P* = 0.362
Ovarian cancer	6	4.525 (1.763–11.618)	0.002	random	*I*^*2*^ = 92.8%; *P* < 0.001
Cervical cancer	2	2.524 (1.152–5.529)	0.021	random	*I*^*2*^ = 66.5%; *P* = 0.084
***DFS***					
Overall	8	2.917 (1.715–4.963)	<0.001	random	*I*^*2*^ = 83.9%; *P* < 0.001
Breast cancer	4	2.966 (1.997–4.405)	<0.001	fixed	*I*^*2*^ = 0.0%; *P* = 0.948
Ovarian cancer	3	2.817 (0.957–8.292)	0.060	random	*I*^*2*^ = 93.5%; *P* < 0.001
*D***istant metastasis (Yes/No)**					
Overall	8	3.739 (2.558–5.466)	<0.001	fixed	*I*^*2*^ = 0.0%; *P* = 0.619
Breast cancer	7	3.480 (2.342–5.170)	<0.001	fixed	*I*^*2*^ = 0.0%; *P* = 0.710
***Lymph node metastasis (Yes/No)***					
Overall	12	2.696 (1.874–3.879)	<0.001	random	*I*^*2*^ = 53.2%; *P* = 0.015
Breast cancer	6	1.880 (1.433–2.465)	<0.001	fixed	*I*^*2*^ = 38.8%; *P* = 0.147
Ovarian cancer	3	5.328 (1.870–15.175)	0.002	random	*I*^*2*^ = 55.1%; *P* = 0.108
Cervical cancer	2	4.732 (1.922–11.652)	0.001	fixed	*I*^*2*^ = 0.9%; *P* = 0.315

HR: hazard ratio; OR: odds ratios; OS: overall survival; CSS: cancer-specific survival; DFS: disease-free survival.

**Table 4:** Inclusion criteria for eligible studies.

Study types	observational studies (prospective or retrospective)
Language	English, Chinese
Databases	PubMed, Wiley Online Library, Science Direct, EMBASE, Cochrane Central Register of Controlled Trials, ISI Web of Science, China National Knowledge Infrastructure (CNKI) and Wanfang database
Tumor type	female reproduction malignancies
Sample	tissue
Sample size	>50
Tumor stage	unlimited
Examined method	IHC
Follow-up	unlimited
Outcome	OS, CSS, DFS and metastasis
Analysis	presenting the HRs/ORs value and its 95% CIs, Kaplan-Meier survival curves or relevant data to calculate HRs/ORs

IHC: immunohistochemistry; OS: overall survival; CSS: cancer-specific survival; DFS: disease-free survival.

## References

[b1] Torre L. A. . Global cancer statistics, 2012. CA Cancer J Clin. 65, 87–108 (2015). 25651787 10.3322/caac.21262

[b2] Chen W. . Cancer statistics in China, 2015. CA Cancer J Clin. 66, 115–132 (2016). 26808342 10.3322/caac.21338

[b3] Brown D. M. . Metadherin, a cell surface protein in breast tumors that mediates lung metastasis. Cancer Cell. 5, 365–374 (2004). 15093543 10.1016/s1535-6108(04)00079-0

[b4] Su Z. Z. . Identification and cloning of human astrocyte genes displaying elevated expression after infection with HIV-1 or exposure to HIV-1 envelope glycoprotein by rapid subtraction hybridization, RaSH. Oncogene. 21, 3592–3602 (2002). 12032861 10.1038/sj.onc.1205445

[b5] Li J. . Astrocyte elevated gene-1 is a novel prognostic marker for breast cancer progression and overall patient survival. Clin Cancer Res. 14, 3319–3326 (2008). 18519759 10.1158/1078-0432.CCR-07-4054

[b6] Meng F. . Clinical significance of astrocyte elevated gene-1 expression in human epithelial ovarian carcinoma. Int J Gynecol Pathol. 30, 145–150 (2011). 21293286 10.1097/PGP.0b013e3181ffd2f7

[b7] Huang K. . High expression of astrocyte elevated gene-1 (AEG-1) is associated with progression of cervical intraepithelial neoplasia and unfavorable prognosis in cervical cancer. World J Surg Oncol. 11, 297 (2013). 24256614 10.1186/1477-7819-11-297 PMC3866971

[b8] Song H. . Expression of astrocyte elevated gene-1: a novel marker of the pathogenesis, progression, and poor prognosis for endometrial cancer. Int J Gynecol Cancer. 20, 1188–1196 (2010). 21495225 10.1111/igc.0b013e3181ef8e21

[b9] Luo Y. . Astrocyte Elevated Gene-1 as a Novel Clinicopathological and Prognostic Biomarker for Gastrointestinal Cancers: A Meta-Analysis with 2999 Patients. PLoS One. 10, e0145659 (2015). 26710214 10.1371/journal.pone.0145659 PMC4692396

[b10] Yang C. . Metadherin is required for the proliferation, migration, and invasion of esophageal squamous cell carcinoma and its meta-analysis. Transl Res. 166, 614–626 e612 (2015). 26051629 10.1016/j.trsl.2015.05.004

[b11] Wan L. . Pleiotropic roles of AEG-1/MTDH/LYRIC in breast cancer. Adv Cancer Res. 120, 113–134 (2013). 23889989 10.1016/B978-0-12-401676-7.00004-8

[b12] Liu P. . miR-26a suppresses tumour proliferation and metastasis by targeting metadherin in triple negative breast cancer. Cancer Lett. 357, 384–392 (2015). 25434799 10.1016/j.canlet.2014.11.050

[b13] Ward A. . Re-expression of microRNA-375 reverses both tamoxifen resistance and accompanying EMT-like properties in breast cancer. Oncogene. 32, 1173–1182 (2013). 22508479 10.1038/onc.2012.128

[b14] Hu G. . MTDH activation by 8q22 genomic gain promotes chemoresistance and metastasis of poor-prognosis breast cancer. Cancer Cell. 15, 9–20 (2009). 19111877 10.1016/j.ccr.2008.11.013 PMC2676231

[b15] Su P. . Immunohistochemical analysis of Metadherin in proliferative and cancerous breast tissue. Diagn Pathol. 5, 38 (2010). 20565850 10.1186/1746-1596-5-38 PMC2906416

[b16] Li C. . Significance of AEG-1 expression in correlation with VEGF, microvessel density and clinicopathological characteristics in triple-negative breast cancer. J Surg Oncol. 103, 184–192 (2011). 21259255 10.1002/jso.21788

[b17] Li C. . AEG -1 overexpression: a novel indicator for peritoneal dissemination and lymph node metastasis in epithelial ovarian cancers. Int J Gynecol Cancer. 21, 602–608 (2011). 21543927 10.1097/IGC.0b013e3182145561

[b18] Li C. . Elevated expression of astrocyte elevated gene-1 (AEG-1) is correlated with cisplatin-based chemoresistance and shortened outcome in patients with stages III-IV serous ovarian carcinoma. Histopathology. 60, 953–963 (2012). 22372608 10.1111/j.1365-2559.2012.04182.x

[b19] Long M. . Overexpression of astrocyte-elevated gene-1 is associated with cervical carcinoma progression and angiogenesis. Oncol Rep. 30, 1414–1422 (2013). 23835593 10.3892/or.2013.2598

[b20] Dong R. . miR-145 inhibits tumor growth and metastasis by targeting metadherin in high-grade serous ovarian carcinoma. Oncotarget. 5, 10816–10829 (2014). 25333261 10.18632/oncotarget.2522 PMC4279412

[b21] Du C. . MTDH mediates trastuzumab resistance in HER2 positive breast cancer by decreasing PTEN expression through an NFkappaB-dependent pathway. BMC Cancer. 14, 869 (2014). 25417825 10.1186/1471-2407-14-869 PMC4254009

[b22] Li C. . Astrocyte elevated gene-1: a novel independent prognostic biomarker for metastatic ovarian tumors. Tumour Biol. 35, 3079–3085 (2014). 24234336 10.1007/s13277-013-1400-0

[b23] Tokunaga E. . Overexpression of metadherin/MTDH is associated with an aggressive phenotype and a poor prognosis in invasive breast cancer. Breast Cancer. 21, 341–349 (2014). 22903204 10.1007/s12282-012-0398-2

[b24] Song E. . Astrocyte elevated gene-1 promotes progression of cervical squamous cell carcinoma by inducing epithelial-mesenchymal transition via Wnt signaling. Int J Gynecol Cancer. 25, 345–355 (2015). 25695541 10.1097/IGC.0000000000000381

[b25] Tan L. . [Expression and clinical significance of MTDH and VEGF in triple-negative breast cancer]. Zhonghua Zhong Liu Za Zhi. 37, 827–832 (2015). 26887512

[b26] Zhou B. . Overexpression of astrocyte-elevated gene-1 is associated with ovarian cancer development and progression. Mol Med Rep. 11, 2981–2990 (2015). 25483832 10.3892/mmr.2014.3056

[b27] Wang Y. . AEG-1 as a predictor of sensitivity to neoadjuvant chemotherapy in advanced epithelial ovarian cancer. Onco Targets Ther. 9, 2385–2392 (2016). 27143933 10.2147/OTT.S102648 PMC4844502

[b28] Jin P. . Expression of metadherin gene in metastatic breast cancer and its clinical significance. Academic Journal of Pla Postgraduate Medical School. 34, 133–136 (2013).

[b29] Higgins J. P. . Quantifying heterogeneity in a meta-analysis. Stat Med. 21, 1539–1558 (2002). 12111919 10.1002/sim.1186

[b30] Meng X. . Drug resistance mediated by AEG-1/MTDH/LYRIC. Adv Cancer Res. 120, 135–157 (2013). 23889990 10.1016/B978-0-12-401676-7.00005-X PMC3967868

[b31] Meng X. . Knockdown of MTDH sensitizes endometrial cancer cells to cell death induction by death receptor ligand TRAIL and HDAC inhibitor LBH589 co-treatment. PLoS One. 6, e20920 (2011). 21687633 10.1371/journal.pone.0020920 PMC3110819

[b32] Kong X. . Inhibition of metadherin sensitizes breast cancer cells to AZD6244. Cancer Biol Ther. 13, 43–49 (2012). 22336587 10.4161/cbt.13.1.18868

[b33] Zhang N. . The oncogene metadherin modulates the apoptotic pathway based on the tumor necrosis factor superfamily member TRAIL (Tumor Necrosis Factor-related Apoptosis-inducing Ligand) in breast cancer. J Biol Chem. 288, 9396–9407 (2013). 23408429 10.1074/jbc.M112.395913 PMC3611009

[b34] Zhao Y. . Metadherin regulates radioresistance in cervical cancer cells. Oncol Rep. 27, 1520–1526 (2012). 22367022 10.3892/or.2012.1692

[b35] Zhang X. . Astrocyte elevated gene-1 induces breast cancer proliferation and invasion through upregulating HER2/neu expression. Chin Med J (Engl). 124, 3546–3550 (2011). 22340175

[b36] Zhao Y. . Metadherin mediates lipopolysaccharide-induced migration and invasion of breast cancer cells. PLoS One. 6, e29363 (2011). 22195048 10.1371/journal.pone.0029363 PMC3241708

[b37] Lee S. G. . Astrocyte elevated gene-1 activates cell survival pathways through PI3K-Akt signaling. Oncogene. 27, 1114–1121 (2008). 17704808 10.1038/sj.onc.1210713

[b38] Meng X. . Cytoplasmic Metadherin (MTDH) provides survival advantage under conditions of stress by acting as RNA-binding protein. J Biol Chem. 287, 4485–4491 (2012). 22199357 10.1074/jbc.C111.291518 PMC3281628

[b39] Wan L. . MTDH-SND1 interaction is crucial for expansion and activity of tumor-initiating cells in diverse oncogene- and carcinogen-induced mammary tumors. Cancer Cell. 26, 92–105 (2014). 24981741 10.1016/j.ccr.2014.04.027 PMC4101059

[b40] Bhutia S. K. . Astrocyte elevated gene-1 induces protective autophagy. Proc Natl Acad Sci USA. 107, 22243–22248 (2010). 21127263 10.1073/pnas.1009479107 PMC3009793

[b41] Zhang J. . Metadherin confers chemoresistance of cervical cancer cells by inducing autophagy and activating ERK/NF-kappaB pathway. Tumour Biol. 34, 2433–2440 (2013). 23595222 10.1007/s13277-013-0794-z

[b42] Krishnan R. K. . Quantitative analysis of the TNF-alpha-induced phosphoproteome reveals AEG-1/MTDH/LYRIC as an IKKbeta substrate. Nat Commun. 6, 6658 (2015). 25849741 10.1038/ncomms7658 PMC4396366

[b43] Xu C. . MTDH mediates estrogen-independent growth and tamoxifen resistance by down-regulating PTEN in MCF-7 breast cancer cells. Cell Physiol Biochem. 33, 1557–1567 (2014). 24854844 10.1159/000358719

[b44] Tierney J. F. . Practical methods for incorporating summary time-to-event data into meta-analysis. Trials. 8, 16 (2007). 17555582 10.1186/1745-6215-8-16 PMC1920534

[b45] DerSimonian R. . Meta-analysis in clinical trials revisited. Contemp Clin Trials. 45, 139–145 (2015). 26343745 10.1016/j.cct.2015.09.002 PMC4639420

[b46] Mantel N. . Statistical aspects of the analysis of data from retrospective studies of disease. J Natl Cancer Inst. 22, 719–748 (1959). 13655060

[b47] Higgins J. P. . Measuring inconsistency in meta-analyses. BMJ. 327, 557–560 (2003). 12958120 10.1136/bmj.327.7414.557 PMC192859

[b48] Liberati A. . The PRISMA statement for reporting systematic reviews and meta-analyses of studies that evaluate healthcare interventions: explanation and elaboration. BMJ. 339, b2700 (2009). 19622552 10.1136/bmj.b2700 PMC2714672

