# Supplementary material for: Association of MTDH immunohistochemical expression with metastasis and prognosis in female reproduction malignancies: a systematic review and meta-analysis
Source: Sci Rep. 2016 Dec 5;6:38365. doi: 10.1038/srep38365 (PMC5137005; doi:10.1038/srep38365)
Supplement: Supplementary Information [file srep38365-s1.pdf]

Association of MTDH immunohistochemical expression with metastasis and prognosis in female reproduction malignancies: a systematic review and meta-analysis

Yongbin Hou[1], Lihua Yu[1], Yonghua Mi[1], Jiwang Zhang[1], Ke Wang[1], Liyi Hu[1,2*].

1 Department of clinical laboratory, Affiliated Yongchuan Hospital of Chongqing Medical University, Chongqing 402160, China
2 Department of CIK treatment laboratory, Affiliated Yongchuan Hospital of Chongqing Medical University, Chongqing 402160, China

*Corresponding author:
Email: hlyhhy@163.com

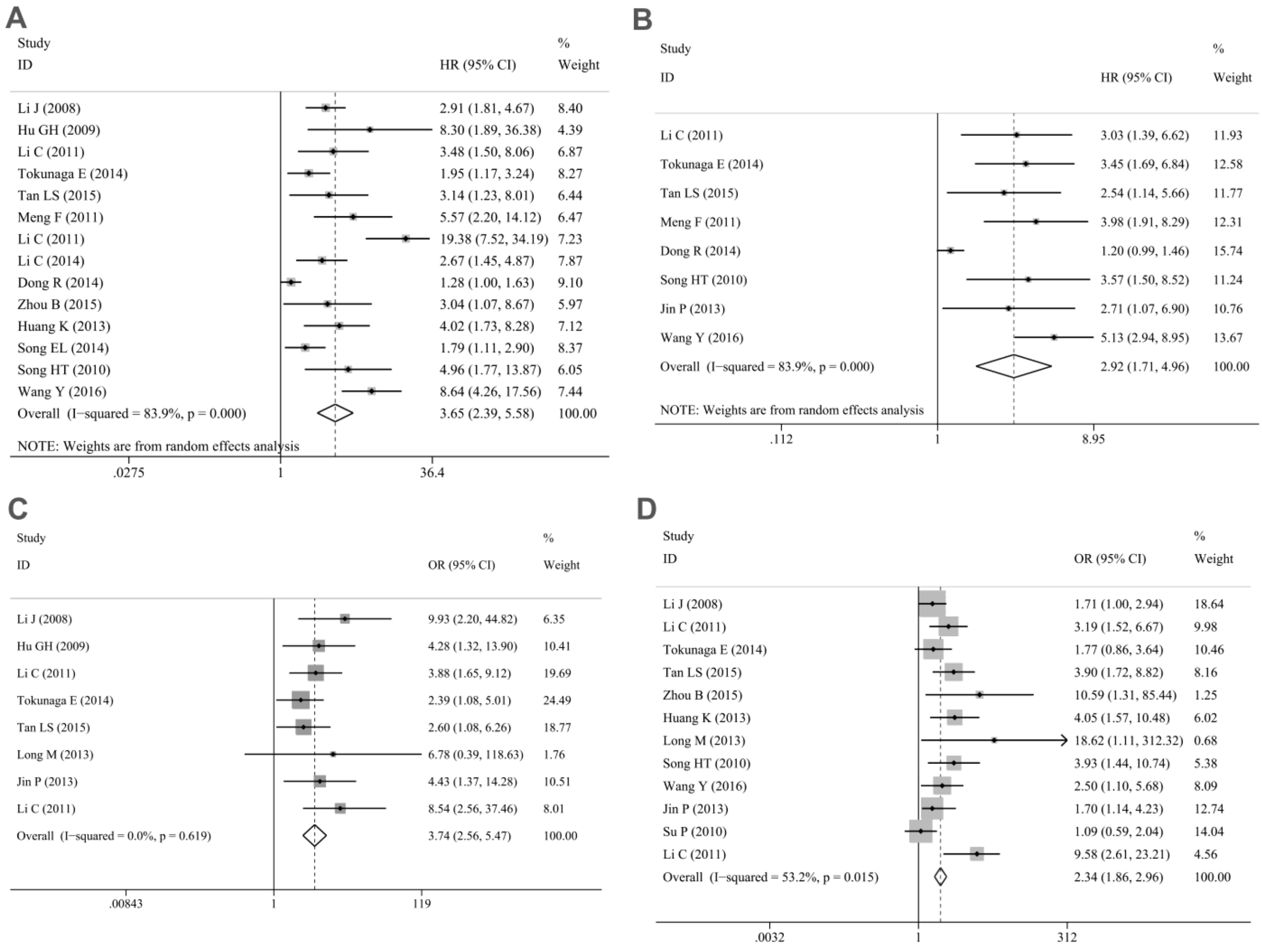

Figure S1. Forest plots of meta-analysis for the association of MTDH with meta-analysis and prognosis in all female reproduction malignancies. (A) mortality; (B) DFS; (C) distant metastasis; (D) lymph node metastasis.

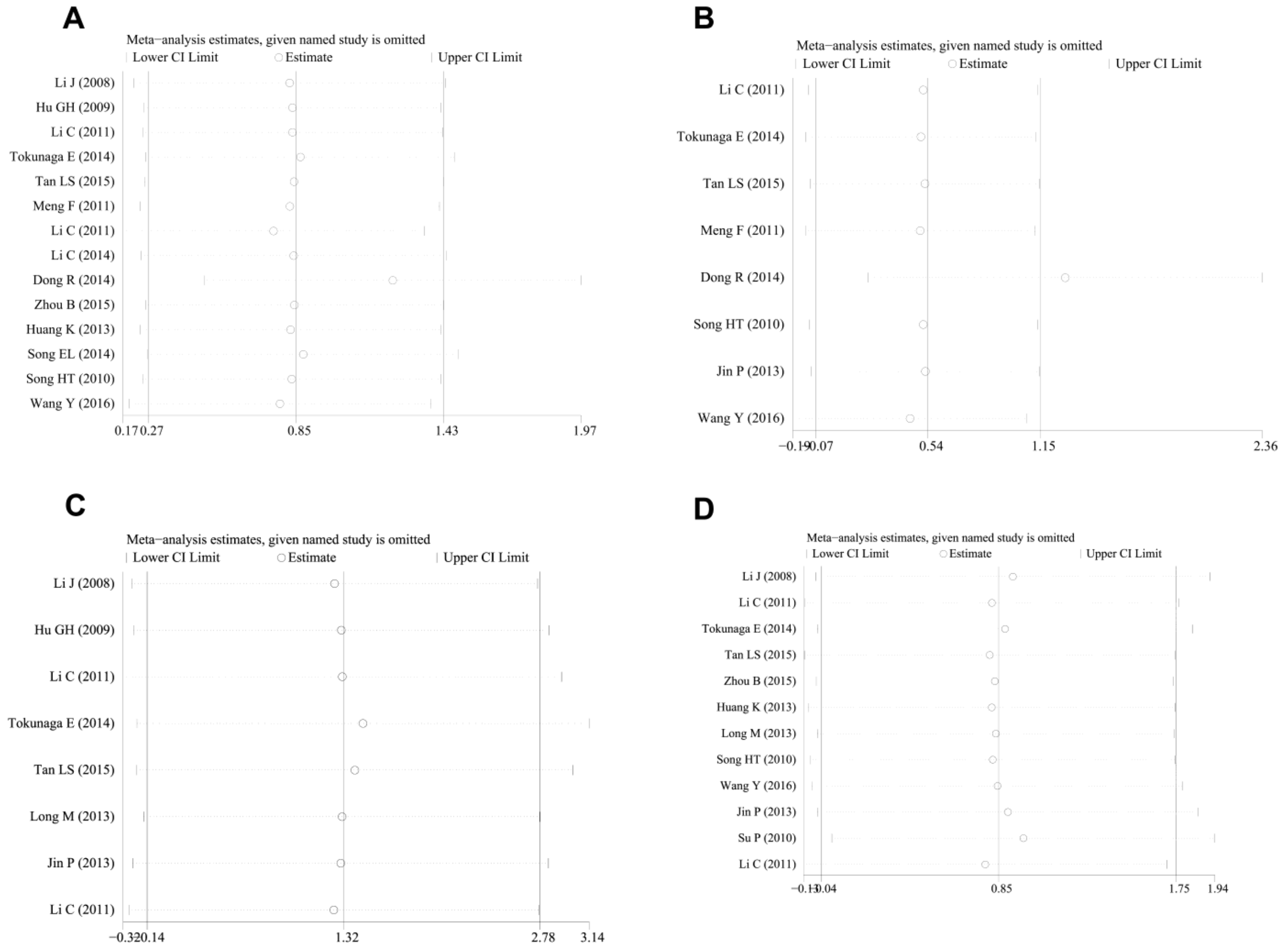

Figure S2. Sensitivity analyses of all included studies in female reproduction malignancies. (A) mortality; (B) DFS; (C) distant metastasis; (D) lymph node metastasis.

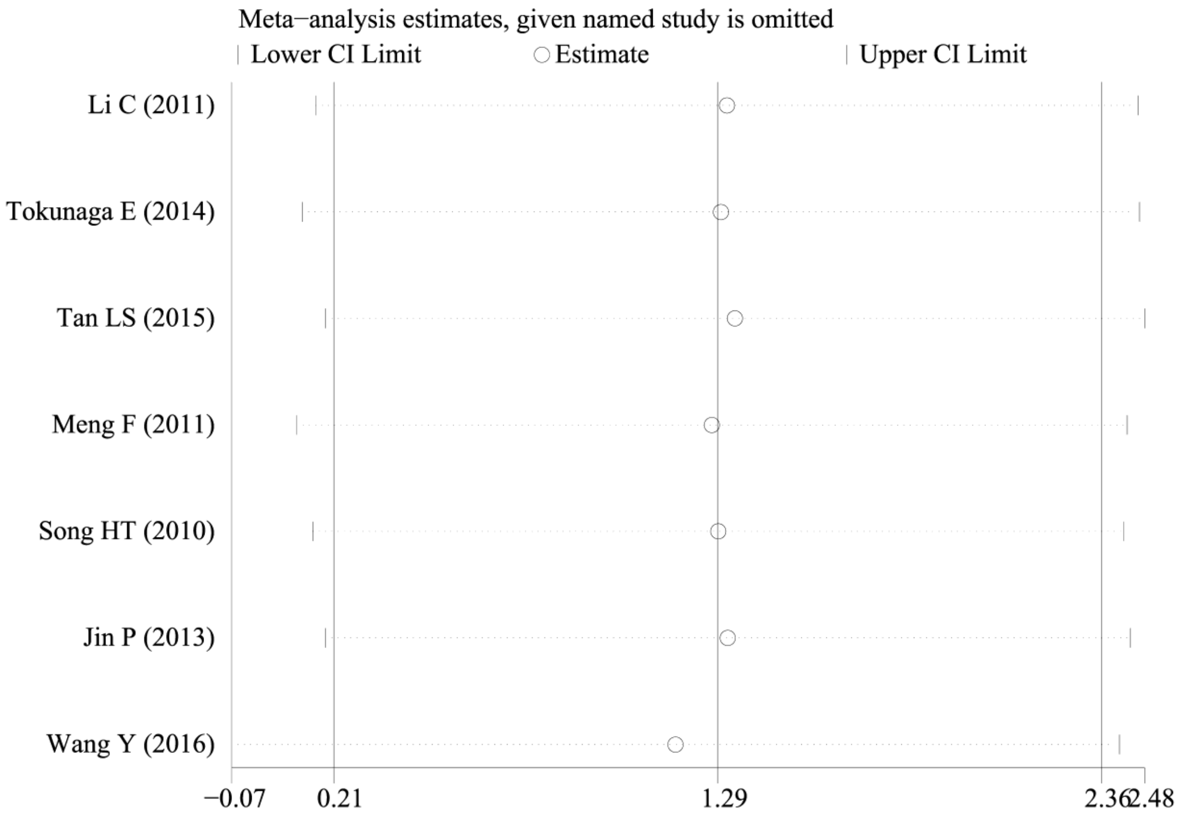

Figure 3S. Sensitivity analyses for DFS after excluding study published by Dong R et al.

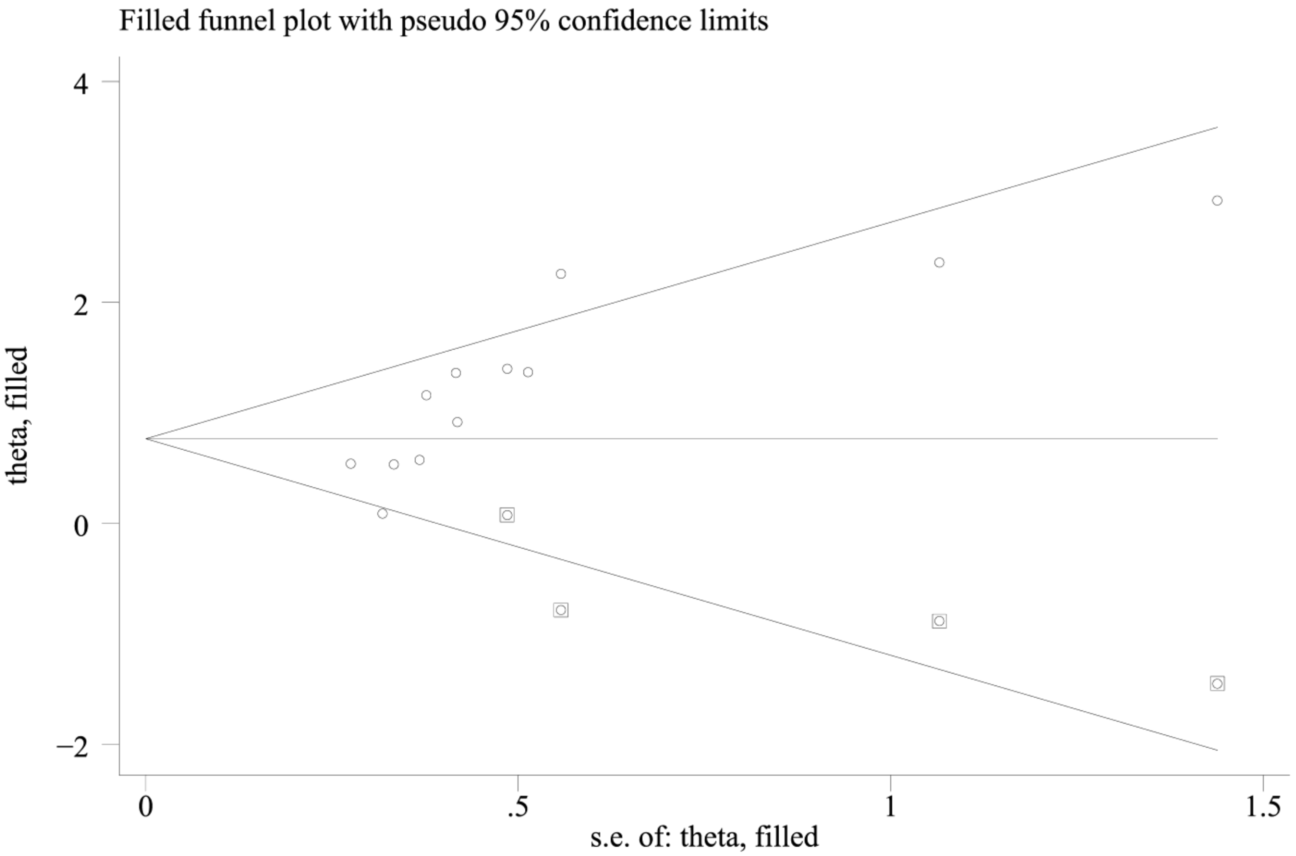

Figure S4. Funnel plot for lymph node metastasis group after enrolling missing studies.
